# Effect of circadian rhythm and menstrual cycle on physical performance in women: a systematic review

**DOI:** 10.3389/fphys.2024.1347036

**Published:** 2024-04-24

**Authors:** Michaela Beníčková, Marta Gimunová, Ana Carolina Paludo

**Affiliations:** ^1^ Department of Physical Activities and Health Sciences, Faculty of Sports Studies, Masaryk University, Brno, Czechia; ^2^ Department of Sport Performance and Exercise Testing, Faculty of Sports Studies, Masaryk University, Brno, Czechia

**Keywords:** biological rhythm, daytime, ovarian cycle, endurance, strength, female

## Abstract

The literature has extensively investigated potential factors influencing women’s performance, including the menstrual cycle and the circadian rhythm. However, review articles exploring the combined influence of both factors remain lacking in the literature. The study aimed to systematically review the literature on the relationship between the circadian rhythm and menstrual cycle on physical performance in women. The review followed the PRISMA guidelines. The search was performed on the Web of Science, PubMed, Scopus, SPORTDiscus, and Google Scholar databases. Of the 1205 records identified, four articles met the inclusion criteria. From four articles, two studies found a significant interaction between the time of day and phase of the menstrual cycle with physical performance. The isometric strength increased in the afternoon in the mid-luteal phase (*versus* the morning in the mid-luteal phase, *p* < 0.05), and the maximum cycling power was higher in the afternoon in the mid-follicular phase (*versus* the morning in the mid-follicular phase, *p* < 0.01). Our findings suggest that the time of day and the menstrual cycle phase affect physical performance, with the daytime potentially having a more significant influence. However, the results should be interpreted cautiously due to the limited studies and diverse methodologies used to monitor the menstrual cycle phase. This review reinforces the need for further investigation to understand better the combined effects of the circadian rhythm and menstrual cycle on women’s physical performance.

**Systematic Review Registration:**

https://www.crd.york.ac.uk/prospero/display_record.php?RecordID=380965, identifier CRD42022380965.

## 1 Introduction

Physical performance (PP) is a complex interplay of multifaceted factors, including the physiological parameters, neuromuscular function, energy expenditure, and psychological elements required to execute a motor task (Astrand, 1968). These tasks involve different components, such as strength, endurance, and power. The investigation into factors influencing PP, particularly within physical activity and sports settings, has been extensive, focusing on understanding the nuances within the female population. Women’s PP has been related to their circadian rhythms (CR), which, in turn, interact with their menstrual cycle (MC), resulting in a complex physiological context that can impact some aspects of physical capabilities. The CR, lasting approximately 24 h, governs diverse bodily processes, including hormone secretion, sleep-wake patterns, basal body temperature regulation, and cognitive and PP (Postolache et al., 2020). Each phase of the CR signifies peaks or falls in different physiological and behavioral functions, significantly influencing factors such as reaction times, physical strength, and endurance (Youngstedt, Elliott, and Kripke, 2019).

Regulated by a central pacemaker in the suprachiasmatic nuclei and influenced by external cues like light, CRs synchronize biological processes with environmental cycles, including daily activities such as exercise (Mirizio et al., 2020; Ayala et al., 2021). Individual variations in CRs, known as chronotypes, dictate when the PP peak occurs. For instance, individuals with evening chronotypes may excel in late-day activities due to their natural alignment with circadian peaks. At the same time, those with morning tendencies may perform best in the early afternoon. Additionally, some individuals do not fall into extreme chronotypes and exhibit intermediate preferences, effectively standing apart from these two distinct chronotype categories. These individuals are categorized under ‘neither’ or ‘intermediate’ chronotypes (Adan et al., 2012). Studies indicate that maximal PP typically occurs in the afternoon, coinciding with peaks in body temperature, cardiovascular functions, and metabolic activities (Hammouda et al., 2013; Aloui et al., 2017; Bellastella et al., 2019). However, this pattern can differ in women due to the physiological changes during the MC.

The MC spans 21–35 days and adds another layer of complexity to the relationship between CRs and PP. Governed by hormonal fluctuations orchestrated by the hypothalamus-pituitary-ovarian axis, the MC consists of distinct phases, each characterized by a specific hormonal environment (Cook, Kilduff, and Crewther, 2018; Paludo et al., 2022). The MC is divided into two main phases by ovulation: the follicular and the luteal phases. The follicular phase lasts 7–21 days and begins with the early follicular phase (EFP), characterized by low sex hormone concentrations. EFP aligns roughly with menstruation or menstrual bleeding, part of the uterine cycle (Hawkins and Matzuk, 2008). It is further subdivided into the mid-follicular phase (MFP), with increasing estrogen concentrations, and the late follicular phase (LFP), where estrogen concentrations peak. Following this, the ovulatory phase occurs, marked by a short duration where estrogen concentrations fall after the luteinizing hormone (LH) peak and ovulation. The luteal phase, lasting approximately 14 days, includes the early luteal phase (ELP) with increasing progesterone concentrations, the mid-luteal phase (MLP) with peak progesterone, and the second, smaller peak in estrogen concentrations and late luteal phase (LLP) with decreasing concentrations of both ovarian hormones (Elliott-Sale et al., 2021). These hormonal changes affect some aspects of a woman’s physiology, including sleep patterns, motivation, and perceived PP (Carmichael et al., 2021). Notably, fluctuations in estrogen, progesterone, and testosterone levels throughout the MC can influence strength, power, thermoregulation, and substrate utilization, potentially altering PP capacities (Smith et al., 2002; Carmichael et al., 2021).

While research has extensively examined the individual impacts of CRs and the MC on PP, the combined influence remains largely unexplored. Bridging this gap in understanding is crucial for optimizing training and competition strategies for female athletes, considering the intricate interplay between hormonal fluctuations and CRs. By synthesizing existing research and identifying avenues for further investigation, a comprehensive understanding of these intertwined influences can pave the way for tailored training programs and personalized performance optimization strategies in women. Therefore, the purpose of the present systematic review was to summarize the effects of the CR and MC on PP.

## 2 Materials and methods

A systematic review was performed according to the guidelines of the Preferred Reporting Items for Systematic Reviews and Meta-Analyses (PRISMA) updated in 2020 (Page et al., 2021). The protocol was registered in PROSPERO with the identification number CRD42022380965.

### 2.1 Eligibility criteria

PECO criteria were used to evaluate the eligibility of the studies. Population = P: (i) eumenorrheic women between 18 and 40 years old; (ii) not taking hormonal contraceptives; (iii) free of any menstrual-related dysfunctions. Exposure = E: (i) at least two daytimes (e.g., 6:00 and 18:00 h); (ii) minimally two different phases of the menstrual cycle (e.g., follicular and luteal phase). Comparator = C: physical performance (example: strength, power, or endurance tests) (i) at different times of the day; (ii) and during the menstrual phases. Outcomes = O: change or maintenance of physical performance in (i) different daytimes; (ii) and menstrual phases.

### 2.2 Data sources and search strategy

A search strategy was performed in Web of Science, PubMed, Scopus, SPORTDiscus, and Google Scholar databases in December 2022. The search terms with Boolean operators were used in the databases of Web of Science, PubMed, Scopus, and SPORTDiscus (online Supplementary Table S1). In Google Scholar, the following terms with Boolean operators were used due to the limited number of characters: (“circadian rhythm” OR “chronotype” OR “diurnal rhythm”) AND (“menstrual cycle” OR “menstruation”) AND (“sport” OR “athletic” OR “athlete” OR “physical activity” OR “exercise” OR “performance”).

An additional data search was performed in January 2024 to ensure the inclusion of the most recent studies. The search followed the same initial criteria, using the same keywords and databases to identify articles published from January 2023 to January 2024. The decision was made to include the latest research findings on the topic.

### 2.3 Study selection and data extraction

**FIGURE 1:**
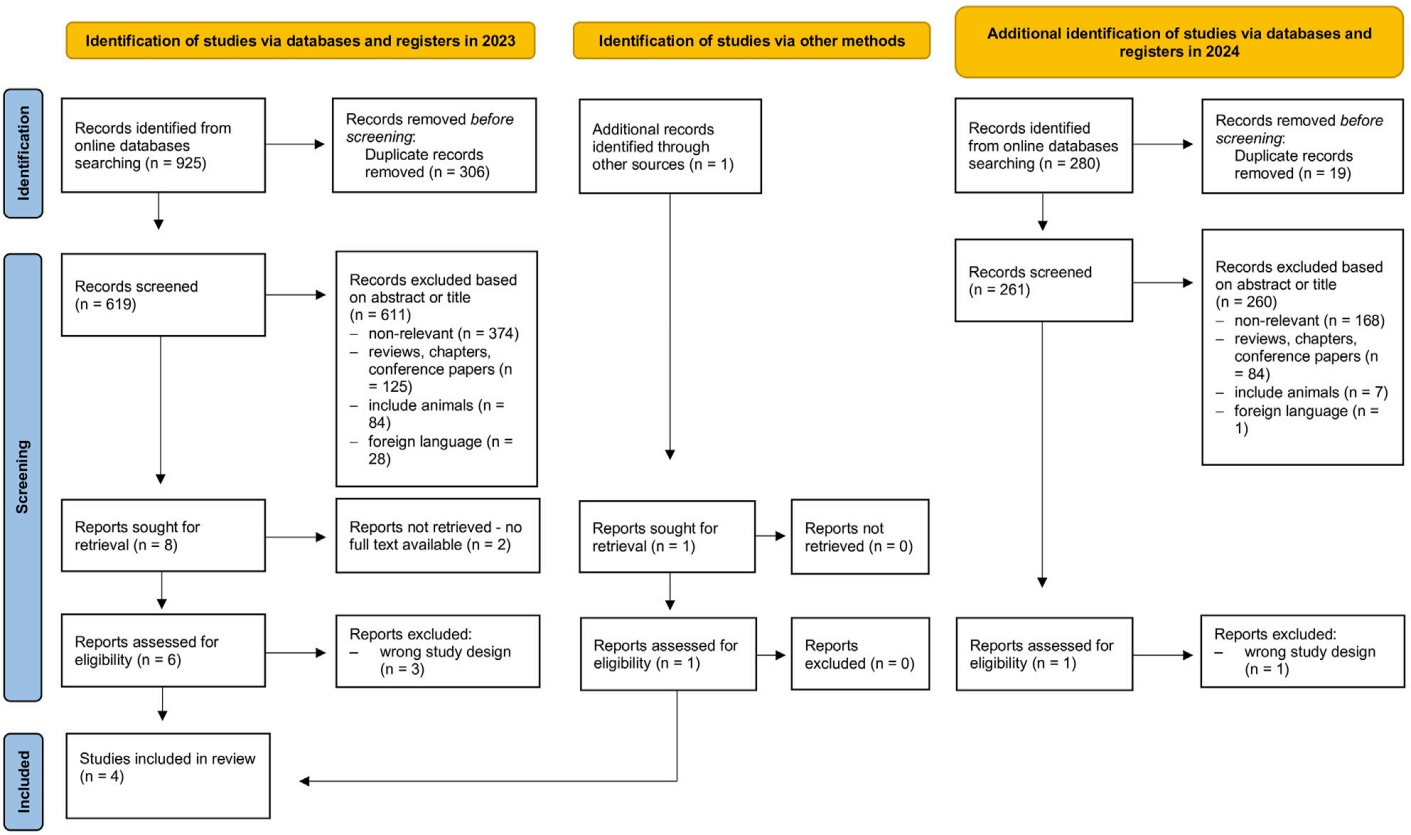
PRISMA flow diagram of the study selection process.

All articles from the databases were imported into Rayyan systematic review software to continue the selection process (Ouzzani et al., 2016). Duplicates, non-English articles, reviews, conference papers, books, and book chapters were excluded by one researcher (MB). Two researchers screened, excluded, or independently accepted the remaining articles (MB, MG). A third person (supervisor, ACP) consulted any disagreement between researchers. The PRISMA flow diagram summarizes the study selection process (Figure 1).

One researcher (MB) extracted data using Microsoft Excel to organize the study’s primary outcomes. Two tables with information about (i) the sample characteristics and methods and (ii) the measurement of the PP and results were developed.

### 2.4 Studies methodological quality

The methodological quality of the included studies was conducted independently by two researchers (MB, MG) using the Downs and Black scale (Downs and Black, 1998). This scale consists of a checklist that comprises quality of reporting, internal and external validity, and statistical power (Downs and Black, 1998). A binary score was used for all items, in which 0 = no/unable to determine (UTD); 1 = yes. The original checklist includes 27 questions, nine irrelevant for accepted studies. This modification has been used in previous articles (Meignié et al., 2021; Paludo et al., 2022), which had a similar approach (see online Supplementary Table S2). Based on these earlier studies, the evaluation of the included studies was converted to percentages and classified as follows: <45.4%, “poor” methodological quality; 45.4%–61.0%, “fair” methodological quality; and >61.0%, “good” methodological quality (Meignié et al., 2021; Paludo et al., 2022). A disagreement about the appraisal was resolved by discussion.

## 3 Results

### 3.1 Literature search

A total of 925 articles were found in the researched databases. After the removal of duplicates (n = 306), 619 studies were screened based on title and abstract, in which 124 articles were excluded due to not meeting the criteria established (e.g., review, conference paper, or book), 84 studies were conducted with animals, 28 published in a foreign language, and 374 were not relevant for our review. Two studies were removed because no full text was available, and three articles had an inadequate study design.

One study was found via another search method (accidental find). This study used different search terms for the expression of CR, met the eligibility criteria, and was accepted for our systematic review. Therefore, we again checked databases using this search term (“moment of day”). However, no relevant studies were included. Four studies were included in the systematic review (Figure 1).

The complementary search performed in January 2024 identified 280 new records, and after the removal of duplicates, no additional studies met the eligibility criteria for inclusion in the review. It reaffirms the findings of the initial search of four studies.

### 3.2 Methodological quality

All studies included were classified using the modified Downs and Black checklist (Downs and Black, 1998) as having “good” methodological quality. Percentage scores for methodological quality were 66.7% (Birch and Reilly, 2002), 68.8% (Giacomoni and Falgairette, 1999; Tounsi et al., 2018) to 75.0% (Bambaeichi et al., 2004).

### 3.3 Outcomes

#### 3.3.1 Studies characteristics

The main characteristics of the four included articles are summarized in Table 1. The studies presented a sample size ranging from eight participants (Bambaeichi et al., 2004) to 11 (Giacomoni and Falgairette, 1999; Tounsi et al., 2018). The studies evaluated high-level soccer players (Tounsi et al., 2018), physical education students (Giacomoni and Falgairette, 1999), moderate physically active females (Birch and Reilly, 2002) and sedentary females (Bambaeichi et al., 2004). The studies used different criteria of selection and the most common were regular MC; non-use of hormonal contraception for at least 4 (Giacomoni and Falgairette, 1999; Bambaeichi et al., 2004), 6 (Tounsi et al., 2018) or 12 (Birch and Reilly, 2002) months; free of any sleep disorder (Bambaeichi et al., 2004) or not to be pregnant or breastfeeding within the previous 4 years (Birch and Reilly, 2002).

**TABLE 1:** Sample characteristics, menstrual cycle, and circadian rhythm measurement.

First author, year	Participants characteristics						Menstrual cycle		Circadian rhythm		
	*N*	Population	Age (y)	Body weight (kg)	Height (m)	Training/week (h)	Methods	Phase	Chronotype	Methods	Time of day (h)
Tounsi (2018)	11	high-level soccer players	21.18 ± 3.15	59.02 ± 7.59	1.63 ± 0.05	N/A	Serum progesterone levels	early follicular	N/A	N/A	7:30
								late follicular			
								luteal phase			17:30
Bambaeichi (2004)	8	sedentary females	30 ± 5	66.26 ± 4.6	1.63 ± 0.06	N/A	BBT and a home ovulation kit	menstruation	intermediate (n = 6)	Chronotype was assessed from the questionnaire. Rectal temperature was used as a marker of the circadian phases	6:00
								mid-follicular			
								ovulation	moderately morning type (n = 1)		
								mid-luteal			18:00
								late luteal	moderately evening type (n = 1)		
Birch (2002)	10	moderate physically active females	24 ± 3	58.4 ± 6.9	N/A	30 min/day for 5 days or more per week	BBT and moliminal symptom analysis	mid-follicular	N/A	The oral temperature was used as a marker of the circadian phases	6:00
								mid-luteal phase			18:00
Giacomoni (1999)	11	physical education students	22.6 ± 2.7	59.7 ± 5.4	1.661 ± 0.085	N/A	Serum progesterone levels	mid-follicular	N/A	Rectal temperature was used as a marker of the circadian phases	9:00
								mid-luteal			14:00
											18:00

The evaluation of chronotype was limited to a single study (Bambaeichi et al., 2004), despite all articles included in this review assessing PP on at least 2 days occasions (morning and afternoon). Circadian phases were evaluated using rectal temperature (Giacomoni and Falgairette, 1999; Bambaeichi et al., 2004) or oral temperature (Birch and Reilly, 2002) as markers.

The participants were observed during at least 2 MC phases (follicular and luteal). The MC phase was established by the day of the MC and confirmed by basal body temperature (Birch and Reilly, 2002; Bambaeichi et al., 2004), serum progesterone levels (Giacomoni and Falgairette, 1999; Tounsi et al., 2018), moliminal symptoms (Birch and Reilly, 2002) or by urine luteinizing hormone (Bambaeichi et al., 2004).

Different tests were used to assess PP, focusing on strength and endurance performance during the MC and the day, for example, Repeated Shuttle-Sprint Ability Test, Yo-Yo Intermittent Recovery Test, Five Jump Test, or maximal voluntary isometric contraction.

#### 3.3.2 Circadian rhythm and physical performance

Two articles identified a variation in strength and speed endurance performance across the day. In the study by Tousi et al. (2018), the authors found a significant improvement in strength performance in high-level soccer players, measured by the Five-Jump test, in the afternoon compared to the morning (*p* = 0.001). Additionally, an improvement in speed endurance performance was observed in the repeated shuttle-sprint ability test performance in the afternoon compared to the morning (*p* = 0.001). Likewise, in the Bambaeichi et al. (2004) study, maximal voluntary isometric contraction of knee extensors under electrical stimulation was significantly increased in the afternoon by 2.6% compared to the morning in sedentary females (*p* < 0.05).

#### 3.3.3 Menstrual cycle and physical performance

The relationship between the MC phase and strength performance was identified in one single study (Bambaeichi et al., 2004). Isokinetic peak torque of knee extensors at 3.15 rad s^-1^ was significantly higher at ovulation than in EFP (76.8 ± 5.7 vs. 73.2 ± 5 N m) in sedentary females and also higher in the MLP (76.9 ± 5 N m) than both EFP (73.2 ± 5 N m) and MFP (72.1 ± 5 N m). To isokinetic peak torque of knee flexors at 1.05 rad s^-1^ the performance was better during the ovulation (75.5 ± 5 N m) compared to the MFP (68.6 ± 4.2 N m) and MLP (68.4 ± 4.6 N m). The ovulation phase seems the most favorable for isometric contraction of knee flexors. During ovulation, strength performance was significantly better than in the MFP and MLP (75.5 ± 5 *versus* 68.6 ± 4.2 and 68.4 ± 4.6 N m).

#### 3.3.4 Circadian rhythm, menstrual cycle, and physical performance

The effects of the interaction of the time of day and MC phase were found in two studies. The maximal isometric lifting strength (MILS) differed significantly during the day and MCs, with a considerable increase in performance in the afternoon during the MLP compared to morning in the MLP in moderate physically active females (*p* < 0.05) (Birch and Reilly, 2002). On the other hand, the maximal cycling power performance was significantly higher in the afternoon during the MFP compared to morning during the MFP in physical education students (9:00 vs. 14:00 h, *p* < 0.05; 9:00 vs. 18:00 h, *p* < 0.01) (Giacomoni and Falgairette, 1999).

The remaining parameters, such as anaerobic endurance, mentioned in Table 2, were not significantly influenced by the time of day or MC phase (Birch and Reilly, 2002; Tounsi et al., 2018).

**TABLE 2:** Main outcomes.

First author, year	Physical performance measurement		Results	
	Strength	Endurance	Strength	Endurance
Tounsi (2018)	Lower limb explosive power was measured by the Five Jump Test (5JT)	Speed endurance was measured using the Repeated Shuttle-Sprint Ability Test (RSSA)	↑ absolute performance in 5JT at 17:30 compared to 7:30 h	↔ YYIRT1 by ToD or MC.
				↑ RSSA at 17:30 compared to 7:30 h
		Anaerobic endurance was measured by Yo-Yo Intermittent Recovery Test (YYIRT)	↔ 5JT by MC.	↔ RSSA by MC.
			↔ ToD and MC interaction in 5JT.	↔ ToD and MC interaction in 5JT.
Bambaeichi (2004)	PTfle and PText at 1.05 and 3.14 rad/s	–	↑ MILS under ES at 18:00 by 2.6% than at 06:00 h	–
			↑ PText at 3.14 rad/s at O than at M, at mid-L than at M and mid-F	
	MVC of knee EXT and FLE were measured at 0 rad/s and 60°of knee flexion		↑ PTfle at 1.05 rad/s at O than mid-F and mid-L	
			↑ MVCfle at O than at mid-F and mid-L	
	MILS of knee EXT were performed with percutaneous electrical twitches		↔ between ToD and MC. MC had a more significant effect than ToD	
Birch (2002)	MILS at knee height	Endurance capability at 45% MILS.	↑ MILS by 8.3% at 18:00 h in the mid-L	↔ between ToD and MC in endurance capability at 45% MILS.
			↔ MILS by ToD in the mid-F	
Giacomoni (1999)	Maximal cycling power (Pc) by force-velocity test	–	↑ Pc in the afternoon in the mid-F	–
			↔ Pc by ToD in the mid-L	

↔, no significant interaction/difference; ↑, significant increase/improvement; 5JT, Five Jump Test; RSSA, Repeated Shuttle-Sprint Ability Test; YYIRT1, Yo-Yo Intermittent Recovery Test level 1; PText, peak torque of knee extensors; PTfle, peak torque of knee flexors; MVC, maximal voluntary isometric contraction; EXT, extensors; FLE, flexors; ES, electrical stimulation; MILS, maximal isometric lifting strength; Pc, maximal cycling power; M, menstruation; O, ovulation; F, follicular phase; L, luteal phase; MC, menstrual cycle phase; ToD, time-of-day.

## 4 Discussion

This systematic review aimed to examine the effect of the interaction between CR and the MC on PP. Initially, the review provided an overview of the potential relationship between CR, MC, and PP. The findings suggest that strength and endurance performance in women may vary depending on the time of day and MC phase. Studies have described increased strength and speed endurance performance during the afternoon. Furthermore, ovulation seems to be the most favorable phase for strength performance, with improvements in isokinetic peak torque and isometric contraction of knee flexors. However, it is worth emphasizing that this finding regarding the ovulation’s optimal phase for performance enhancement is based on findings from a single study, warranting further research for confirmation.

### 4.1 Circadian rhythm and physical performance

A few studies have already focused on the effect of CR on PP; however, most of them have investigated the male population. Ayala et al. (2021) summarized the possible impact of CR on PP in a systematic review, in which only two out of 36 included studies investigated females, and eight investigated both males and females. In the remaining 26 studies, only men or individuals of undefined sex were examined. Similarly, the gender gap was found by Mirizio et al. (2020) when the authors invested the effect of day on short-duration maximal exercise performance. The author demonstrated that from 66 included articles, two studies included exclusively females and ten studies of both sexes. The remaining samples were made up solely of males, and sex was not mentioned.

Based on previous studies, PP seems to peak during the afternoon, together with the body’s basal temperature (Mirizio et al., 2020; Ayala et al., 2021). Additionally, the individual’s chronotype plays a vital role in this phenomenon. Morning chronotypes typically experience their performance peak in the early afternoon, while evening chronotypes experience it later in the afternoon (Ayala et al., 2021). This difference is likely attributed to differences in body temperature patterns, with morning chronotypes exhibiting an earlier peak in the afternoon than evening chronotypes (Lack et al., 2009). Despite the relevance of the chronotype in understanding performance patterns, our systematic review found limited data, with only one included study assessing chronotype without directly correlating it with PP (Bambaeichi et al., 2004).

In general, based on previous literature and the included studies, the PP usually peaks in the afternoon (Giacomoni and Falgairette, 1999; Birch and Reilly, 2002; Bambaeichi et al., 2004; Tounsi et al., 2018; Mirizio et al., 2020; Ayala et al., 2021). More specifically, Ayala et al. (2021) observed a peak of PP between 16:30–18:30. Mirizio et al. (2020) found a PP peak between 16:00–20:00 and studies included in our systematic review have achieved similar results, with the highest PP at 17:30 (Tounsi et al., 2018) or 18:00 (Giacomoni and Falgairette, 1999; Birch and Reilly, 2002; Bambaeichi et al., 2004).

On the other hand, the drop in performance occurred in the morning (Giacomoni and Falgairette, 1999; Birch and Reilly, 2002; Bambaeichi et al., 2004; Tounsi et al., 2018; Mirizio et al., 2020; Ayala et al., 2021). The decrease in performance can be attenuated by using some techniques. For example, Mirizio et al. (2020) recommend an active warm-up protocol or warm-up while listening to neutral or high-tempo music, exposure to warm and humid climate conditions, regular training in the morning for at least 4 weeks, or intermittent fasting conditions.

There is also speculation that peak performance, especially during testing, tends to occur when the training and testing times align (Bruggisser et al., 2023). Therefore, it is prudent to determine the typical training times of the participants who take part in the studies to avoid bias in the performance outcomes.

### 4.2 Menstrual cycle and physical performance

There is growing attention on the influence of the MC on exercise response in sportswomen. Since 2020, about five systematic review studies have examined the relationship between MC and different components of PP (Blagrove et al., 2020; McNulty et al., 2020; Thompson et al., 2020; Meignié et al., 2021; Romero-Parra et al., 2021). Some of the results of these systematic reviews were summarized in an umbrella review published in 2023 (Colenso-Semple et al., 2023), demonstrating the lack of significant influence of the MC phase on acute strength performance or adaptations to resistance exercise training, underlining the need for more rigorous and methodologically consistent research in this area. However, none of these articles focused on the interaction effect between the MC and CR on PP.

Our systematic review suggests improvement in strength performance during the ovulation phase compared to the luteal and follicular phases, according to a study by Bambaeichi et al. (2004). The remaining studies did not consider the ovulation phase (Giacomoni and Falgairette, 1999; Birch and Reilly, 2002; Tounsi et al., 2018), examining the EFP, LFP, and luteal phase (Tounsi et al., 2018), MFP and MLP (Birch and Reilly, 2002) or MFP and MLP (Giacomoni and Falgairette, 1999).

Generally, PP appeared to be relatively stable throughout the MC (Blagrove et al., 2020; McNulty et al., 2020; Meignié et al., 2021; Colenso-Semple et al., 2023) but may be trivially impaired during the EFP (McNulty et al., 2020). This phenomenon can be related to the hormonal characteristic of this phase, particularly with lower estrogen and progesterone concentration levels. Estrogen, known for its anabolic properties and influence on metabolic processes such as glycogen uptake and utilization, is essential in protecting muscles against oxidative stress and damage during physical activity. Additionally, its neuroexcitatory impact, which includes enhancing voluntary muscle activation, suggests that higher estrogen levels in phases other than the EFP could potentially improve muscular and exercise performance. In contrast, the lower estrogen levels during the EFP do not confer these performance benefits (McNulty et al., 2020; Carmichael et al., 2021). Besides, EFP is marked by menstrual bleeding, symptoms, discomfort, pain, and decreased vigor (Paludo et al., 2022). However, the overall impact on performance during this phase is considered minor, emphasizing the need for individualized consideration of exercise performance across different phases of the MC. Uncertainty in the results of this study can be due to the poor quality of the included studies, various methodologies, and a small number of participants.

### 4.3 Circadian rhythm, menstrual cycle, and physical performance

Previous sections of this review have explored the influences of CR and the MC effect on PP. Yet, it is crucial to consider their combined effects. The interplay between these two biological processes holds promise for a more holistic comprehension of the optimal conditions for women’s PP.

Distinctively, the MC exerts its influence primarily via hormonal fluctuations. Certain phases, such as ovulation, may boost strength performance due to hormonal profiles (Bambaeichi et al., 2004). Conversely, the CR predominantly modulates performance through physiological variations across the day, with performance peaks commonly observed during the afternoon (Mirizio et al., 2020; Ayala et al., 2021).

The intersection of these cycles suggests potential windows within a female cycle wherein performance could be optimized. For instance, the synchronization of the afternoon (a circadian peak) with specific menstrual phases hints at periods of enhanced performance. Nevertheless, existing literature provides scarce direct evidence regarding this intersection. Studies exploring these connections, such as those by Birch and Reilly (2002) and Giacomoni and Falgairette (1999), offer preliminary insights rather than definitive conclusions. A significant research gap exists in investigations explicitly focusing on the combined effects of these cycles.

### 4.4 Limitations of the study

Although this systematic review presents the effect of CR and MC on PP for the first time, there are limitations to bear in mind. The lack of studies investigating the current topic, the small sample size, and the different tests and methodologies to assess menstrual phase, strength, and endurance performance measures evaluated among the studies could be significant limitations to the generalization of the results. Considering the lack of a standard for measuring PP or quantifying MC phases and chronotypes, the study’s findings should be interpreted cautiously.

We recommend that future studies on this topic include larger sample sizes of women with different levels of physical activity and sports disciplines to address these limitations. Standardized methods should be used to investigate the effects of time of day and MC phase on PP. Additionally, studies should address potential confounding factors, such as chronotype, hormonal levels, and other physiological or lifestyle factors, that might influence the relationships between CRs, MC phases, and PP. Understanding the interactions between the time of day, MC phase, and PP can better support the optimization of training and competition strategies for women, considering the complex interactions of their biological rhythms to help them reach their full potential.

## 5 Conclusion

This systematic review is the first to examine the combined effect of circadian rhythm and menstrual cycle on physical performance in women. In summary, the interaction effect of the time of day and phase of the menstrual cycle on physical performance was found in only two studies. While the isometric strength increased in the afternoon in the mid-luteal phase, the maximum cycling power was higher in the afternoon in the mid-follicular phase. These observations are drawn from a minimal number of studies. Our findings suggest that the time of day and the menstrual cycle influence physical performance, with the former potentially having a more significant impact when considered separately. However, interpreting this conclusion warrants caution due to the limited number of studies examining the effect of these factors on physical performance and the variation in methods for tracking menstrual cycle phases. Further research is needed to investigate and clarify the interaction effect of circadian rhythm and menstrual cycle on physical performance.

## Data Availability

The original contributions presented in the study are included in the article/Supplementary Material, further inquiries can be directed to the corresponding author.
