# Supplementary material for: Effect of circadian rhythm and menstrual cycle on physical performance in women: a systematic review
Source: Front Physiol. 2024 Apr 24;15:1347036. doi: 10.3389/fphys.2024.1347036 (PMC11076705; doi:10.3389/fphys.2024.1347036)
Supplement: Supplementary file 1 [file Table1.DOCX]

Table 1: Combination of search terms

| #1 | Circadian rhythm | | “circadian rhythm“ OR “circadian rhythms“ OR “twenty-four hour rhythm“ OR “twenty four hour rhythm“ OR “diurnal rhythm“ OR “daytime“ OR “day time“ OR “day-time“ OR “time of day“ OR “chronotype“ OR “evening type“ OR “evening-type“ OR “morning type“ OR “morning-type“ OR “neither type“ OR “circadian typology” OR “morningness” OR “eveningness” |
| --- | --- | --- | --- |
| #2 | Menstrual cycle | | “menstrual cycle“ OR “menstrual phase” OR “follicular phase“ OR “luteal phase“ OR “menstruation“ OR “ovulation“ |
| #3 | Physical performance | Sport generally | "athletic performances“ OR “sports performance“ OR "exercise“ OR "physical activity“ OR "physical exercise“ OR “sport“ OR “athletics“ OR “athletic“ OR “athlete“ OR “athletes“ OR “college athlete“ OR “professional athlete“ OR “elite athlete“ OR "well-trained“ OR “athletic performances” OR “performance, athletic” OR “performances, athletic” OR “sports performance” OR “performance, sports” OR “performances, sports” OR “sports performances” |
|  |  | Strength | "muscle strength“ OR "strength performance“ OR "muscular performance“ OR “strength“ OR “power“ OR “torque“ OR “force“ OR “neuromuscular“ OR “isometric“ OR “isokinetic“ OR “skeletal muscle“ OR “eccentric“ OR “concentric“ OR “anaerobic“ OR “anaerobic power“ OR “anaerobic capacity“ OR “plyometric“ OR “plyometry“   \|  \|  \| \| --- \| --- \| |
|  |  | Endurance | “aerobic“ OR “aerobic power“ OR “aerobic capacity“ OR “endurance“ OR “endurance power“ OR “endurance capacity“ OR “endurance performance“ OR “stamina” |
|  |  | Speed | “sprint” OR” agility” OR “high-intensity“ OR “velocity“ OR “speed“ |
|  |  | Coordination | “coordination” OR “reaction time” OR “rhythm abilities” OR “balance” OR “postural stability” |
|  |  | Flexibility | “flexibility” OR “range of motion“ OR “laxity” OR “stretching” OR “RoM” |
|  |  | Discipline | “alpine skiing” OR “aquatics” OR “archery” OR “artistic gymnastics” OR “artistic swimming” OR “athletics” OR “badminton” OR “baseball” OR “basketball” OR “beach volleyball” OR “boxing” OR “biathlon” OR “bobsleighing” OR “canoe” OR “cross-country skiing” OR “curling” OR “cycling” OR “diving” OR “equestrian” OR “fencing” OR “figure skating” OR “football” OR “freestyle skiing” OR “golf” OR “gymnastics” OR “handball” OR “hockey” OR “horse riding” OR “ice hockey” OR “judo” OR “karate” OR “kayak” OR “luge” OR “Nordic combined” OR “marathon swimming” OR “mountain bike” OR “pentathlon” OR “rhythmic gymnastics” OR “rugby” OR “running” OR “rowing” OR “sailing” OR “shooting” OR “short track” OR “skateboarding” OR “skeleton” OR “ski jumping” OR “snowboarding” OR “sport climbing” OR “soccer” OR “speed skating” OR “surfing” OR “swimming” OR “table tennis” OR “taekwondo” OR “tennis” OR “track and field” OR “trampoline” OR “triathlon” OR “volleyball” OR “water polo” OR “wrestling” OR “weightlifting” OR “3x3 basketball” OR “softball” OR “BMX racing” OR “BMX freestyle” OR “road cycling” OR “track cycling” |
| **Combination of search terms #1 AND #2 AND #3** | | | |
