# Supplementary material for: Effect of circadian rhythm and menstrual cycle on physical performance in women: a systematic review
Source: Front Physiol. 2024 Apr 24;15:1347036. doi: 10.3389/fphys.2024.1347036 (PMC11076705; doi:10.3389/fphys.2024.1347036)
Supplement: Supplementary file 2 [file Table2.docx]

Table 2: Adjusted Downs and Black Quality Assessment Checklist

| **Reporting** |  |  |  |
| --- | --- | --- | --- |
| 1. *Is* *the* *hypothesis/aim/objective clearly described?*   This item is only rated as a Yes, if both aim/purpose and hypothesis are described. In case the study design does not allow any hypothesis or the direction of the study is so novel that no prior hypothesis can be formed, and this is made clear in the introduction, the item should be rated as a Yes. | YES | NO | UD |
| 1. *Are the main outcomes to be measured clearly described in the Introduction or Methods section?*   If the main outcomes are first mentioned in the Results section, the question should be answered No. |  |  |  |
| 1. *Are the characteristics of the participants included in the study clearly described?*   It was considered if the characteristics about chronotype, menstrual cycle regularity, not use of oral contraception and the menstrual cycle length. It was rated with a YES with the study presenting at least two of these characteristics. |  |  |  |
| 1. *Are the principal confounders compared clearly described?*   If the study report confounders such as oral contraception use or menstrual irregularities during the study the answers will be YES. For a study that does not specify the confounders, the question should be answered as unable to determine (UD). |  |  |  |
| 1. *Are the main findings of the study clearly described?*   In this review, the effect of the menstrual cycle and circadian rhythm should be reported as major findings, so the reader can check the major analyses and conclusions. |  |  |  |
| 1. *Does the study provide estimates of random variability provided for main outcomes?*   In non-normally distributed data the inter-quartile range of results should be reported. In normally distributed data the standard error, standard deviation or confidence intervals should be reported. If the distribution of the data is not described, it must be assumed that the estimates used were appropriate and the question should be answered Yes. If no mean and SD are calculated and only individual items are provided, rate this item as a No. |  |  |  |
| 10. *Have the probability values been reported for main outcomes?*  Does the study present p-value e.g. 0.035 rather than <0.05 for  the  main  outcomes  except where the probability value is less than 0.001? Rate Yes if the study includes this criterion. |  |  |  |
| **External validity** |  |  |  |
| 11. *Were the subjects asked to participate in the study representative of the entire population from which they were recruited?*  The study must identify the source population for participants and describe how the participants were selected. Participants would be representative if they comprised the entire source population, an unselected sample of consecutive participants, or a random sample. Random sampling is only feasible where a list of all members of the relevant population exists. Where a study does not report the proportion of the source population from which the patients are derived, the question should be answered as unable to determine. The source population in our review is defined as female athletes in the respective sports. |  |  |  |
| 12. *Were those subjects who were prepared to participate representative of the entire population from which they were recruited?*  The proportion of those asked who agreed should be stated. Validation that the sample was representative would include demonstrating that the distribution of the main confounding factors was the same in the study sample and the source population. |  |  |  |
| **Internal validity – bias** |  |  |  |
| 16. *If any of the results of the study were based on “data dredging”, was this made clear?*  Any analyses that had not been planned at the outset of the study should be clearly indicated. If no retrospective unplanned subgroup analysis were reported, then answer YES. |  |  |  |
| 17. *In trials and cohort studies, do the analyses adjust for different lengths of follow‐up of patients, or in case‐control studies, is the time period between the intervention and outcome the same for cases and controls?*  For this question, it was considered the menstrual phase determination. Studies that combined the day of menstrual cycle together with the ovulation determinants such as urine LH strip or hormones concentration. The answer YES for those that use the approach, and NO if the study uses only the day of the menstrual cycle. |  |  |  |
| 18. *Were the statistical tests used to assess the main outcomes appropriate?*  The statistical techniques used must be appropriate to the data. Where little statistical analysis has been undertaken but where there is no evidence of bias, the question should be answered yes. If the distribution of the data (normal or not) is not described it must be assumed that the estimates used were appropriate and the question should be answered yes. |  |  |  |
| 20. *Were the menstrual cycle phase and perceptual responses measures used accurate (valid and reliable)?*  Studies have to report on the reliability and validity of the tools to estimate the menstrual cycle phases (e.g., urine LH strips, days of the cycle...) and the circadian rhythm (e.g., body temperature…). For studies that refer to other work that demonstrates the outcome measures are accurate, the question should be answered as Yes. Both, pilot testing or referring to the work of others is sufficient to rate this item as a Yes. |  |  |  |
| 21. *Were the participants in different intervention groups (trials and cohort studies) or were the cases and controls (case-control studies) recruited from the same population?*  The source population in this review is defined as female athletes in the respective sports. If the athletes were recruited from different sports modalities, the answer was NO. |  |  |  |
| 22. *Were study participants in different intervention groups (trials and cohort studies) or were the cases and controls (case-control studies) recruited over the same period of time?*  It was considered with the athletes who started the study in the same period as a YES or if the studies collected data from athletes in different periods of time the answer was NO. For a study that does not specify the time period over which athletes were recruited, the question should be answered as unable to determine (UD). |  |  |  |
| 25. *Was there an adequate adjustment for confounding in the analyses from which the main findings were drawn?*  This item should be rated as a Yes, if the criteria for verification of the menstrual cycle are explicitly stated. Verification of menstrual cycle phase should contain one of the following criteria: 1) different days during the menstrual cycle with different hormones concentration (ex: day 1 and day 15), 2) verification of ovulation phase using urine LH strips, 3) ovarian hormone concentration during the phases. The same applies for circadian rhythms. |  |  |  |
| 26. *Were losses of patients to follow‐up taken into account?*  If the numbers of athletes` losses are not reported, the question should be answered as unable to determine. If the proportion lost was too small to affect the main findings, the question should be answered yes. |  |  |  |
| **Power** |  |  |  |
| 27. *Did the study have sufficient power to detect a clinically important effect where the probability value for a difference is due to chance is less than 5%?*    Sample size calculation performed = yes |  |  |  |

Yes (1 point), No (0 points), UD = unable to determine (0 points).
